# Spin polarization of excitons in organic multiferroic composites

**DOI:** 10.1038/srep28656

**Published:** 2016-06-23

**Authors:** Shixuan Han, Liu Yang, Kun Gao, Shijie Xie, Wei Qin, Shenqiang Ren

**Affiliations:** 1 School of Physics, State Key Laboratory of Crystal Materials, Shandong University, Ji Nan, 250100, China; 2 Department of Mechanical Engineering and Temple Materials Institute, Temple University, Philadelphia, PA 19122, USA; 3 College of Physics and Optoelectronics, Taiyuan University of Technology, Taiyuan, 030024, China

## Abstract

Recently, the discovery of room temperature magnetoelectricity in organic charge transfer complexes has reignited interest in the multiferroic field. The solution processed, large-area and low cost organic semiconductor materials offer new possibilities for the functional all organic multiferroic devices. Here we report the spin polarization of excitons and charge transfer states in organic charge transfer composites by using extended Su-Schrieffer-Heeger model including Coulomb interaction and spin-flip effect. With the consideration of spin polarization, we suggest a possible mechanism for the origin of excited ferromagnetism.

In recent years, multiferroic materials have received significant consideration for its potential applications in spintronics, optoelectronics, thermoelectric and sensors^1^. The most widely known inorganic multiferroic materials are based on the perovskite structure, such as BiFeO_3_^2^ and TbMnO_3_^3^. In these materials, the charge, spin, orbital and phonon degrees of freedom are strongly entangled, which lead to substantial advance in condensed matter physics and materials science. In addition to the inorganic counterparts, organic multiferroics has been attracting significant interests^4^,^5^, which offers a new route toward magnetoelectric multiferroics. In 2009, Giovannetti *et al*. first predicted organic multiferroicity in TTF-CA (tetrathiafulvalene-p-chloranil) charge transfer salt through a combination of Ab initio method and model Hamiltonian calculations^6^. Since then, a few organic multiferroics with magnetoelectric coupling have been experimentally discovered^7^,^8^,^9^,^10^,^11^. In this context, polymeric charge transfer crystals, consisting of polythiophene and fullerene, are shown external stimuli controlled magnetization which is resulted from the emergence of spin polarization through intra-molecular or inter-molecular excitons^11^.

Though the remarkable progress and potential impact on all organic multiferroics are achieved, Up to now, the actual mechanism on excited ferromagnetism in organic composites is still infancy. Absence of the corresponding properties in inorganic counterparts indicates that some intrinsic factors of organic semiconductors may play important role. The recent investigation on the spin polarization of charged organic molecules or polymers suggests that the injected charges could induce spontaneous spin polarization and a net magnetic moment could appear in Alq_3_ even it contains no any magnetic elements^12^. Furthermore, it is found that the spin polarization or magnetic moment in a charged thiophene is sensitive to the intrinsic electron-phonon (e-ph) coupling as well as its polymerization^13^,^14^. Therefore, it is expected that an excited state should be spin polarized if the electron and hole are located in different segments of an organic donor-acceptor composite.

In this paper, we select a prototypical example system, organic nanowire polythiophene/fullerene (nw-P3HT/C_60_) composites, for the modeling by using tight-binding approach and study their spin-related excited states.

## Model

In the investigation of organic excited ferromagnetism, electron donors (D) are typically *π*-conjugated polymers or molecules, such as nw-P3HT in this study. The acceptors (A) are usually small molecules or clusters, such as fullerene and its derivatives. For an organic composite composed of donor and acceptor segments, the Hamiltonian consists of three parts,


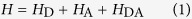


where *j*(*j* = D, A) is the molecule index, *H*_*j*_ describes the corresponding segment. We model it in one-dimensional tight-binding approach^15^,


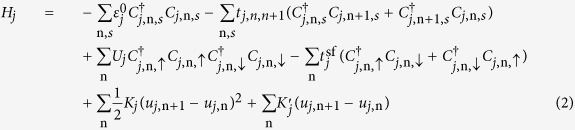



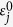
 denotes the on-site energy of *π*-electrons segment *j*, which determines the energy offset between donor and acceptor. The second term in Eq. 2 describes the nearest hopping of *π*-electrons within segments with transfer integral 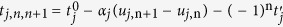. The e-ph coupling is reflected by parameter *α*_*j*_. The third term denotes the electron-electron interaction with Hubbard approach, which is treated within Hartree-Fock approximation 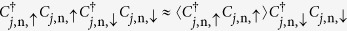 + 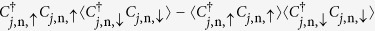. While the small exchange interaction term 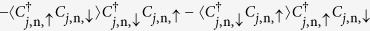 is included in the spin-flip term. The fourth term describes the spin-flip of *π*-electrons with flip integral 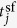. This term is caused by many factors, such as the electron-electron exchange interaction mentioned above, the hyperfine interaction from the hydrogen nuclei^16^, the thermal effect^17^ as well as the spin-related scattering. Due to the low mobility of organic materials, an electron has a long staying time at sites. So we suppose that the spin-flip takes place mostly when it stays at sites. The spin-orbit coupling^18^ causes spin-flip when an electron transfers from one site to another. As we consider the stationary excitons in present work, we neglect it in the present work^14^. The involvement of the third and fourth term destroys the degeneracy of the spin state and the system becomes spin mixing. The fifth term denotes the elastic energy due to the lattice displacement and *K*_*j*_ is the lattice elastic constant. The last term is added to stabilize the boundary of each segment and 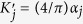
19. All other variables have the normal meaning.

**Figure 1:**
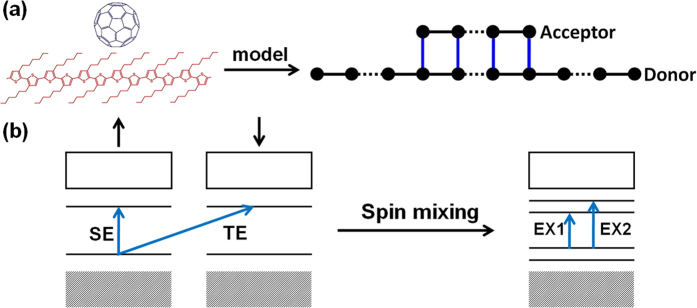
Schematic diagrams of (**a**) P3HT/C_60_ composite and the model, and (**b**) excited transition between HOMO and LUMO before and after spin mixing.

*H*_DA_ denotes the coupling between donor and acceptor,


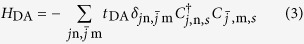


where *t*_DA_ is the intermolecular electron transfer integral from one site in the donor molecule to the corresponding one in the acceptor molecule. Usually *t*_DA_ is much smaller than the intramolecular integral 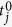. The 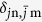 term means that the sum is restricted in the coupling area as shown in Fig. 1(a). We expand the electronic state in the basis of Wannier wave function as,


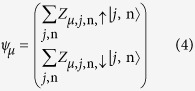


where *Z*_*μ*,*j*,n,*s*_ (*s* = ↑, ↓) means the probability amplitude of state *ψ*_*μ*_ with spin *s* at site n in segment *j*. In this case, each state will become spin mixing with probability 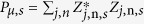 for spin *s*. Then the spin polarization or magnetic moment of the system is given by (in unit of *ħ*/2), 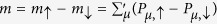, where 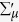 means sum over all the occupied states. Electronic state *ψ*_*μ*_ with energy *ε*_*μ*_ is obtained by solving the Schrodinger equation *Hψ*_*μ*_ = *ε*_*μ*_*ψ*_*μ*_, which is determined by,


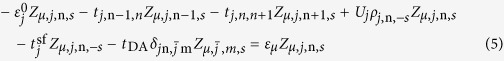


where 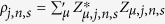. The site displacement *u*_*j*,*n*_ in equilibrium state is given by the equilibrium condition,


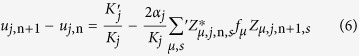


where *f*_*μ*_ is the Fermi distribution.

Eqs 5 and 6 are solved self-consistently as following. Firstly, the eigenvalue equation Eq. 5 is solved with an initial configuration {*u*_*j*,n_}, and then substitute the eigenstates into equation Eq. 6 to get a new configuration. The calculations will be repeated with this new configuration to retain the lowest energy solutions. The parameters are chosen around those of polyacetylene, *t*_*j*_ = 2.5 eV, *α*_*j*_ = 4.1 eV/Å, *K*_*j*_ = 21 eV/Å^2 ^19, and *U*_*j*_ = 1.0 eV, 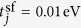
14, 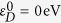, 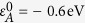 and *t*_*DA*_ = 0.1 eV^20^. Some parameters are changed to discuss their effect on the properties of the system.

## Results and Discussion

In organic semiconductors, an exciton is produced through electron transition from HOMO (highest occupied molecular orbital) to LUMO (lowest unoccupied molecular orbital) by photoexcitation. The transition probability is proportional to the square of the matrix element 〈*ψ*_LUMO_|*H*′|*ψ*_HOMO_〉^21^, where *H*′ denotes the electron-photon interaction. Usually, as the speed of an electron is much lower than light speed in a semiconductor, the electric field of light plays a dominant role during the excitation. Therefore, in the first order approximation, as for transition between spin pure states, the main production of inter-band excitation is the spin singlet exciton (SE, as shown in Fig. 1(b)). While the spin triplet exciton (TE, as shown in Fig. 1(b)) is neglected. However, if *H*′ includes spin related interactions such as spin-flip effect, the production of triplet exciton may increase apparently.

If the electronic state is spin mixing, the transition of TE and SE will evolve into EX1 and EX2 (as shown in Fig. 1(b)). Both of them can take place even *H*′ does not include spin related interactions. For the transition EX1, the matrix element is 〈*ψ*_L1_|*H*′|*ψ*_HOMO_〉 = 〈*ψ*_L1↑_|*H*′|*ψ*_HOMO↑_〉 + 〈*ψ*_L1↓_|*H*′|*ψ*_HOMO↓_〉, where the arrow indicts the spin component of the LUMO (L1 and L2) and the HOMO states. In the following, we will consider all the possible inter-band transition and then check the spin polarization of the excited states.

**Figure 2:**
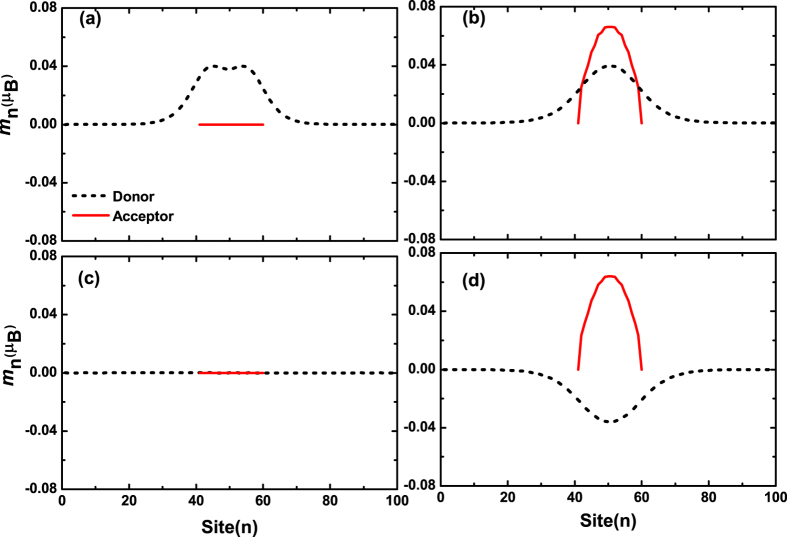
Spin density distributions for intra-molecule exciton EX1 and EX2 (**a**,**c**); and inter-molecule exciton EX1 and EX2 (**b**,**d**).

In an organic charge transfer composite, the photoexciation may take place within one segment (donor or acceptor) to form an intra-molecule exciton or between two segments to form an inter-molecule exciton. If the donor-acceptor coupling is weak, an intra-molecule exciton is stable. Because of the strong e-ph coupling, the excited electron is still bound to the hole in real space. Therefore, the intra-molecule exciton has no net charge distribution, only the lattice distortion. The spin density distribution for intra-molecule exciton in donor is shown in Fig. 2(a,c), where we find that there is a localized spin distribution for EX1, although the exciton is neutral everywhere. The total net magnetic moment is 1.98 *μ*_B_, where *μ*_B_ is the Bohr magneton. The difference between EX1 and the spin pure triplet state (with magnetic moment 2 *μ*_B_) results from the spin mixing. As for EX2, there is no any spin distribution, which corresponds to the spin singlet state.

For an inter-molecule exciton, the situation is very different. An inter-molecule exciton is, in fact, a charge transfer state, which means that either the donor or the acceptor is charged. From our previous investigation^14^, we know that a charged molecule is spin spontaneous polarized. Therefore, it is expected that both the donor and acceptor will be spin polarized after photoexcitation. Because the donor and acceptor are two different materials, the intensity of spin polarization should also be different. The results are shown in Fig. 2(b,d), where all the parameters are the same with that of intra-molecule exciton. For EX1 with one electron is excited, it is found that the donor is spin polarized with magnetic moment 0.91 *μ*_B_. The acceptor has a magnetic moment 0.96 *μ*_B_. The total magnetic moment of EX1 is 1.87 *μ*_B_. For EX2, the donor and acceptor has a magnetic moment of −0.85 *μ*_B_ and 0.93 *μ*_B_, respectively, and thus the total magnetic moment is 0.08 *μ*_B_. The spin polarization of the donor and the acceptor is observed by measuring the electron spin resonance (ESR) spectroscopy in composites shown in our earlier studies^22^,^23^,^24^,^25^, which will be discussed later.

**Figure 3:**
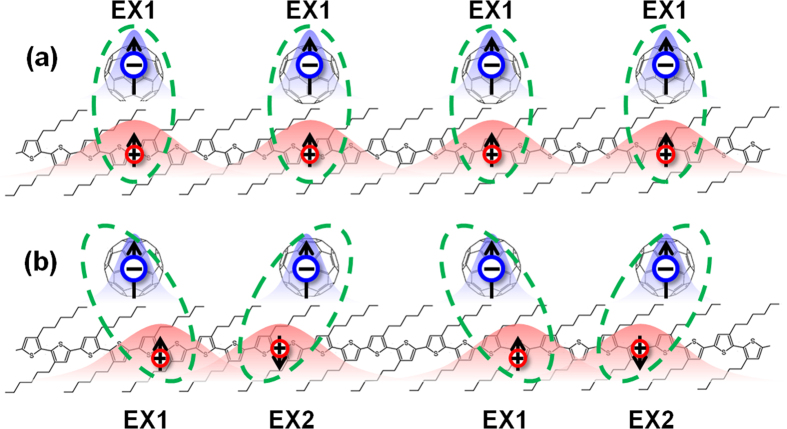
Schematic diagrams of (**a**) only triplet or EX1 excitons in parallel configuration, and (**b**) alternative EX1 and EX2 configuration.

The appearance of spontaneous magnetization for an excited state is crucial to understand the excited magnetism observed in nw-P3HT/C_60_ composites mentioned above. From Fig. 2 or the ref. 11, it clearly indicates that the magnetic moment of charge transfer composites can be more than doubled after the illumination of the sample with a 615 nm laser. Ren *et al*. gave an explanation to their experiment based on the conversion from singlet to triplet excitons. From the present calculation, we can understand it better. Different from the case of spin pure states, where only the triplet exciton has a spin, the singlet exciton has no direct contribution to the magnetization. Because of electron-electron interaction and spin-flip effect, either excited state EX1 or EX2 has a net spin. They both contribute to the excited ferromagnetization. From Stoner theory^26^, we conjecture that it is not easy for two triplet excitons to appear a direct magnetic coupling if the electron-electron interaction is not very strong. However, they may appear an indirect magnetic coupling through an intermediate spin state. In addition, it has been indicated that the excited magnetization has a particular requirement for the conformation of the organic composite^11^. Here we suggest a model as shown in Fig. 3, If the excitons are arranged as in Fig. 3(a), although it is ferromagnetic, the system should be unstable in energy. However, if we consider the involvement of both spin EX1 and EX2, we may design a conformation as shown in Fig. 3(b). The EX1 excitons could take parallel configuration through their coupling with EX2. Although there is no apparent net spin within the polymer donor, the magnetic moment is provided by the spin polarized acceptors. This model is similar to that of organic ferromagnets, such as poly-BIPO^27^,^28^,^29^. The ferromagnetic behavior of the composite is determined by the Heisenberg model 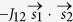, where −*J*_12_ denotes the coupling between EX1 (with spin *s*_1_) and EX2 (with spin *s*_2_). The coupling strength is dependent upon the concentration of excited excitons. Therefore, after illumination, the magnetization of the composite and its characteristic can change apparently.

**Figure 4:**
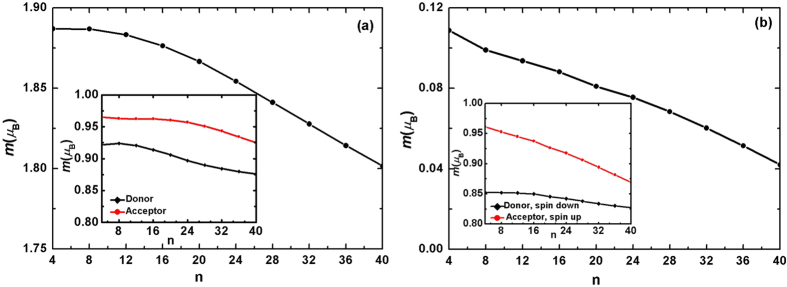
Dependence of the excited magnetic moment on the size of the acceptor. (**a**) is for EX1 and (**b**) is for EX2. Insert: dependence of the magnetic moment of the donor and the acceptor separately on the size of the acceptor.

It has been indicated that, for an isolated charged molecule, the spin polarization is sensitive to the localization of the electronic state. For a small molecule, an electron is confined by the size of the molecule. But for a large molecule, an electron is confined by the strong e-ph coupling. Firstly, we study the effect of the acceptor size on the excited magnetization. The calculation is carried out for one electron excited and the result is shown in Fig. 4. It is found that with the acceptor molecule gets larger, the net magnetic moment decreases rapidly. If we consider the details of the spin polarization, it is found that the polarization of the donor is different from that of the acceptor. In the inset of Fig. 4(a), we show the dependence of the magnetic moment of the donor and the acceptor separately on the size of the acceptor for EX1 (similar for EX2 in Fig. 4(b)). Although only the size of the acceptor molecule changes, it is found that the spin polarization in both donor and acceptor are decreased. This is due to the electronic coupling between electron and hole. While for EX2, the excited electron has higher energy and stronger delocalization than EX1, and the coupling between the electron and hole is weaker than EX1. Thus in the inset of Fig. 4(b), the spin polarization of donor is only slightly decreased.

**Figure 5:**
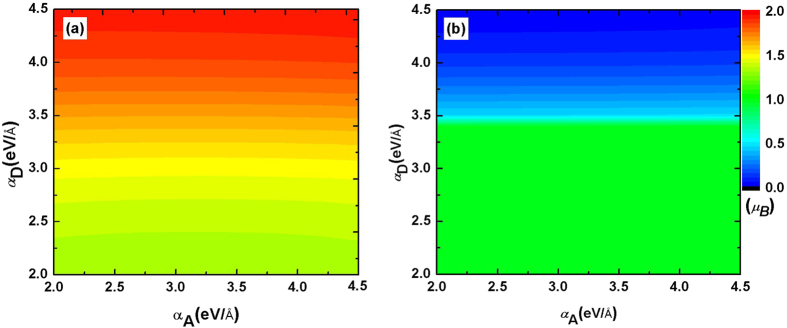
Dependence of the excited magnetic moment on the electron-phonon coupling of donor and acceptor. (**a**) is for EX1 and (**b**) is for EX2.

The donor usually employs a polymer having a chain-like structure. It has been well known that a conjugated polymer has a strong e-ph coupling, which will result in the extra electron (or hole) to form a localized self-trapped state. For example, in cis-polyacetyle, one extra electron (or hole) forms a polaron and two electrons (or holes) form a bipolaron. These excitations are crucial to understand the charge transport in organic semiconducting polymers. Here we study the effect of e-ph coupling of the donor on the magnetism. The calculation is carried out for one electron excited and the result is shown in Fig. 5. Due to the size confinement effect, the electron in acceptor molecule is always localized, regardless of the strength of e-ph coupling *α*_A_. As shown in Fig. 5, the change in *α*_A_ has nothing to do with the total net magnetic moment. But the strength of *α*_D_ can strongly affects the total spin polarization. For the excited state EX1, a strong e-ph coupling could induce a large spin polarization. In the red zone of Fig. 5(a), the net magnetic moment is close to 2 *μ*_B_, which means the spin polarization in donor and acceptor are parallel. While for EX2, the hole in donor and electron in acceptor is spin antiparallel, instead, a small e-ph coupling is conducive to the spin polarization.

Then, let us recognize the ESR spectra measured in composite nw-P3HT/PCBM, SWCNT/C_60_ and nw-P3HT/Au^22^,^23^,^24^,^25^. The ESR peaks are caused by the spin polarization of positive holes in the donor or negative electrons in the acceptor. In nw-P3HT/PCBM, Two separate peaks show that both nw-P3HT and PCBM are spin polarized^22^,^25^. The polarizations result from the transferred charges in each segment. The charges form localized state due to the strong e-ph coupling and become spin polarized. In SWCNT/C_60_, there is only one peak in the ESR spectrum, which comes from C_60_^23^. The transferred charges in SWCNT are extended and they are not spin polarized. While in nw-P3HT/Au, the peak shows that only nw-P3HT is spin polarized^24^. The Au clusters used in experiment have average size 2 nm, which contains hundreds of Au atoms. In this case, the spin polarization due to the confinement is not apparent. Therefore, in nw-P3HT/Au device, there is no peak observed characterizing the Au cluster.

## Conclusions

In this paper, we report a tight-binding model to study the spin polarization of excitons and charge transfer states in organic multiferroic charge transfer composites. It is found that the charge transfer states could be spontaneous spin polarized no matter with the spin states. This is very different from the intra-molecule exciton. We suggest a mechanism that involves low energy excited state EX1 and EX2 which both contribute to the excited magnetization. EX1 excitons could take parallel configuration through their coupling with EX2. We also studied the electron-phonon coupling and size effect on the spin polarization of the excited state EX1 and EX2. In the present work, we consider the spin polarization of one exciton or charge transfer state. We do not calculate the spin couplings between two magnetic moments from different heterojunctions, although these couplings are very important for the presence of ferromagnetism. In addition, many interesting phenomena have been observed in experiments, such as the magnetic anisotropy, ferroelectricity and ferroelasticity *et al*. which need more detailed study.

## Additional Information

**How to cite this article**: Han, S. *et al*. Spin polarization of excitons in organic multiferroic composites. *Sci. Rep.* **6**, 28656; doi: 10.1038/srep28656 (2016).
